# Editorial: New mechanistic insights into mineral regulatory hormones (FGF23/Klotho, PTH, and vitamin D) including genetic and epigenetic pathways

**DOI:** 10.3389/fendo.2024.1361814

**Published:** 2024-01-24

**Authors:** Noriko Ide, Marie Courbebaisse, Rik Mencke, Jun-ichi Hanai

**Affiliations:** ^1^ Division of Bone and Mineral Research, Department of Oral Medicine, Infection, and Immunity, Harvard School of Dental Medicine, Boston, MA, United States; ^2^ Massachusetts General Hospital Krantz Family Center for Cancer Research and Department of Medicine, Harvard Medical School, Charlestown, MA, United States; ^3^ Department of Internal Medicine, Division of Nephrology, Saga University Faculty of Medicine, Saga, Japan; ^4^ Paris Cité University, Physiology Department, European Georges-Pompidou Hospital, Assistance Publique-Hôpitaux de Paris (APHP), Institut National de la Santé et de la Recherche Médicale (INSERM) U1151, Paris, France; ^5^ Department of Pathology and Medical Biology, Division of Pathology, University of Groningen, University Medical Center Groningen, Groningen, Netherlands; ^6^ Department of Neurosurgery, Carl von Ossietzky University Oldenburg, Oldenburg, Germany; ^7^ Division of Nephrology, Division of Interdisciplinary Medicine and Biotechnology (IMBIO), Department of Medicine, Beth Israel Deaconess Medical Center and Harvard Medical School, Boston, MA, United States

**Keywords:** mineral regulatory hormones, mineral ion homeostasis, genetic, epigenetic, DNA Methylation, x-linked hypophosphatemia, cardiac pathology, chromatin-immunoprecipitation (ChIP)-seq

Mineral ion homeostasis is essential for maintaining proper biological functions and cell, tissue, and organ activities. It is crucial to build new evidence for the pathophysiological mechanisms focusing on the regulation and interplay among mineral regulatory hormones, including fibroblast growth factor 23 (FGF23), Klotho, parathormone (PTH), and vitamin D, via integrating new findings from various novel approaches, including techniques that help in elucidating genetic and epigenetic mechanisms.

This Research Topic aimed to introduce new mechanisms of mineral ion regulation that expand on previous research from genetic, epigenetic, and post-translational aspects, all of which are emerging as new potential regulators of mineral metabolism. These new regulators could contribute to various pathogenetic processes.

In details, the review by Portales-Castillo and Simic describes the genetic and epigenetic regulatory mechanisms underlying the actions of PTH, FGF23, Klotho, and 1,25(OH)_2_-vitamin D (calcitriol), which are of clinical relevance. In particular, the importance of several novel mechanisms, such as DNA methylation, mRNA stabilization, and histone modifications in mineral regulation, is addressed. The authors also briefly discuss the role of the parathyroid hormone receptor (PTH1R) in endochondral bone formation and the mediation of PTH and PTH related protein functions. Regarding FGF23 functions, they emphasize the importance of post-translational regulation, such as glycosylation and phosphorylation. In addition, the authors highlight epigenetic changes associated with diseases such as pseudohypoparathyroidism type 1B and rickets, which involve abnormal DNA methylation and decreased expression of certain associated genes.

X-linked hypophosphatemia (XLH) is the most common type of heritable FGF23-related hypophosphatemic rickets. Takashi et al. report a case study of an adult patient with XLH and tertiary hyperparathyroidism. The patient was treated with burosumab, an anti-FGF23 antibody that is clinically available. However, the serum phosphate level and ratio of tubular maximum phosphate reabsorption to glomerular filtration rate (TmP/GFR) remained low. The authors speculated that the high PTH circulating level, due to tertiary hyperparathyroidism, may have suppressed renal phosphate reabsorption despite burosumab treatment. Combination therapy with brosumab and the calcimimetic evocalcet increased the patient’s serum phosphate levels and TmP/GFR. This article suggests that it is important to evaluate the presence of secondary-tertiary hyperparathyroidism in patients whose serum phosphate levels do not increase with burosumab treatment. The authors mention that adult patients with XLH and secondary-tertiary hyperparathyroidism have been excluded from clinical trials evaluating burosumab efficacy. Consequently, further reports or studies are required to evaluate the possible short and long-term effect of burosumab on XLH patients with secondary-tertiary hyperparathyroidism.

In a mini-review article, Nakano et al. summarize the evidence from experimental and clinical studies on associations between FGF23 and cardiac pathologies, such as left ventricular hypertrophy (LVH), heart failure, atrial fibrillation, and myocardial infarction, and discuss potential direct and indirect mechanisms by which FGF23 induces LVH. As a direct effect of FGF23 on LVH, the authors review previous studies that have demonstrated that FGF23 induces cardiac hypertrophy by activating fibroblast growth factor receptor 4 (FGFR4) signaling in cardiomyocytes. This review also highlights some recent studies showing that FGF23 also activates the renin-angiotensin-aldosterone system (RAAS) and induces LVH, and that crosstalk between FGF23 and RAAS contributes to cardiac hypertrophy and fibrosis, suggesting an indirect effect of FGF23 on LVH. Based on findings from published studies, the authors conclude that high FGF23 expression in osteocytes and cardiomyocytes may contribute to the progression of LVH via FGFR4 and angiotensin II receptor type 1 signaling, particularly in the setting of chronic kidney disease.

Pike et al. provide molecular insights into the inter-regulation of mineralotropic hormones PTH, FGF23, and calcitriol, including genetic and epigenetic aspects.

This article underlines the progress made by novel techniques, like chromatin-immunoprecipitation (ChIP)-seq in investigating gene transcription, using the vitamin D receptor (VDR) gene as an example. Furthermore, this article reviews the genomic advances underlying the production of calcitriol by PTH, FGF23, and calcitriol itself, through their actions on the *Cyp27b1* and *Cyp24a1* genes expressed in the renal proximal tubules. In particular, PTH stimulates *Cyp27b1* transcription in a CREB-dependent manner, whereas FGF23 and calcitriol are negative regulators of *Cyp27b1* by interfering with CREB coactivators, with reciprocal effects on *Cyp24a1* expression.

Moreover, the authors discuss the results of their recent studies on the regulation of *Fgf23* transcription using CRISPR-Cas9 gene editing *in vivo.* These studies aimed at identifying the genomic control regions within the *Fgf23* gene locus that mediate the regulatory actions of calcitriol, PTH and phosphate and ultimately to identify unique factors involved in these transcriptional mechanisms. Additionally, the authors describe novel and ongoing experiments that include an acute phosphate response *in vivo*, distinct from the usual chronic response, and begin to detail the mechanism of phosphate-induced *Fgf23* up-regulation in bone.

In summary, this Research Topic provides new insights into the endocrine regulation of mineral ion homeostasis from a wide range of research areas with various approaches, which will lead to the identification of potential new therapeutic targets and agents of mineral imbalance and related diseases. Although there has been a significant increase in research on genetic, epigenetic (DNA methylation, histone modifications, non-coding RNA, and RNA modifications), and post-translational aspects to determine or fill gaps in cellular, molecular, and pathological processes involved in mineral metabolism, further research is needed.

We hope that this Research Topic will be useful to researchers and clinicians who are interested in mineral ion homeostasis as a reference and source of inspiration.

## Author contributions

NI: Writing – original draft, Writing – review & editing. MC: Writing – review & editing. RM: Writing – review & editing. JH: Writing – review & editing.

